# Demographic and clinical characteristics of deaths associated with influenza A(H1N1) pdm09 in Central America and Dominican Republic 2009–2010

**DOI:** 10.1186/s12889-015-2064-z

**Published:** 2015-07-31

**Authors:** Rafael Chacon, Sara Mirza, David Rodriguez, Antonio Paredes, Giselle Guzman, Lourdes Moreno, Cecilia J. Then, Jorge Jara, Natalia Blanco, Luis Bonilla, Wilfrido A. Clara, Percy Minaya, Rakhee Palekar, Eduardo Azziz-Baumgartner

**Affiliations:** Centro de Estudios en Salud, Universidad del Valle de Guatemala, 18 Av. 11-95, zona 15, Vista Hermosa III, Guatemala, Guatemala; Centers for Disease Control and Prevention, Influenza Division, Atlanta, Georgia USA; Ministry of Health, San Salvador, El Salvador; Ministry of Health, Guatemala, Guatemala; Social Security of Costa Rica, San Jose, Costa Rica; Ministry of Health, Panama, Panama; Ministry of Health, Dominican Republic, Dominican Republic; Training of Epidemiology and Public Health Intervention Network, Guatemala, Guatemala; Influenza Group. Pan-American Health Organization, Washington DC, USA

**Keywords:** H1N1 2009, Pandemic influenza deaths, Central america

## Abstract

**Background:**

The demographic characteristics of pandemic influenza decedents among middle and low-income tropical countries are poorly understood. We explored the demographics of persons who died with influenza A (H1N1)pdm09 infection during 2009–2010, in seven countries in the American tropics.

**Methods:**

We used hospital-based surveillance to identify laboratory-confirmed influenza deaths in Costa Rica, El Salvador, Guatemala, Honduras, Nicaragua, Panama and Dominican Republic. An influenza death was defined as a person who died within two weeks of a severe acute respiratory infection (SARI) defined as sudden onset of fever >38 °C, cough or sore-throat, and shortness of breath, or difficulty breathing requiring hospitalization, and who tested positive for influenza A (H1N1)pdm09 virus by real time polymerase chain reaction. We abstracted the demographic and clinical characteristics of the deceased from their medical records.

**Results:**

During May 2009-June 2010, we identified 183 influenza deaths. Their median age was 32 years (IQR 18–46 years). One-hundred and one (55 %) were female of which 20 (20 %) were pregnant and 7 (7 %) were in postpartum. One-hundred and twelve decedents (61 %) had pre-existing medical conditions, (15 % had obesity, 13 % diabetes, 11 % asthma, 8 % metabolic disorders, 5 % chronic obstructive pulmonary disease, and 10 % neurological disorders). 65 % received oseltamivir but only 5 % received it within 48 h of symptoms onset.

**Conclusions:**

The pandemic killed young adults, pregnant women and those with pre-existing medical conditions. Most sought care too late to fully benefit from oseltamivir. We recommend countries review antiviral treatment policies for people at high risk of developing complications.

**Electronic supplementary material:**

The online version of this article (doi:10.1186/s12889-015-2064-z) contains supplementary material, which is available to authorized users.

## Background

Early in the pandemic, the epidemiologic and clinical characteristics of influenza A (H1N1)pdm09 were poorly understood. Pending information about this emerging pathogen, influenza prevention and control policies in the American tropics still targeted persons at risk of developing complications as a result of seasonal influenza disease (e.g. children aged < 5 years, persons with certain chronic medical conditions, and persons aged ≥ 65 years) [1]. For example, health officials worried that physiologic changes during pregnancy and the postpartum period would place women at risk for developing complications as a result of pandemic influenza illness [2–4].

At the start of the pandemic, Central American countries used CDC’s Guidelines for Treatment of Suspected Cases of Influenza A(H1N1)pdm09 [5]. At the end of 2009, Central America countries sought to implement the PAHO treatment recommendations developed in July 2009 [6]. These recommendations prioritized the early use of oseltamivir among children, older adults, pregnant women and people with chronic diseases [7–11]. Health officials used television, print and radio to urge persons presumed to be at high-risk who developed sudden onset fever, cough or sore throat, and all persons who developed danger signs (e.g. respiratory difficulty), to seek timely care [7, 12, 13]. Risk communication messages urged the people to seek medical care, but only Nicaragua, Panamá and Guatemala, explicitly recommended that to people at high risk of developing complications from influenza illness [14–17]. National guidelines recommended to treat patients with oseltamivir when it was most effective (i.e. within 48 h from symptom onset) [1, 7, 9, 11, 18, 19].

WHO also recommended to the countries vaccinate pregnant women and other high risk persons against pandemic influenza [20]. To accomplish this, WHO provided three shipments of donated monovalent influenza A(H1N1)pdm09 vaccine to 4 of the 7 countries in Central America during March 2010. This donation was the only source of influenza vaccine for public health sector and allowed for a vaccination coverage of 14–34 % among the general population in Central America (El Salvador: 2,2 million doses, coverage: 34 %; Guatemala: 1,3 million doses, coverage: 10 %; Honduras: 1,7 million doses, coverage: 24 %; Nicaragua: 750,000 doses, coverage: 14 %) [21].

Pharmaceutical interventions in middle and low income countries of the American tropics were in limited supplies or only available late during the pandemic. In Central America, while in some countries social security coverage is almost universal, in others is as low as 12 %. Investment in health varies between 5-11 % of gross domestic product, the density of beds varies 0.7-2.4 per 1000 inhabitants; physician density 0.4-1.9 per 1000 inhabitants; and rural population of 25–50 % [22]. In this manuscript we explore the impact of the pandemic in Central America and the Dominican Republic through a description of the demographics, clinical characteristics, and oseltamivir treatment of persons who died with laboratory-confirmed influenza A(H1N1)pdm09.

## Methods

### Case identification

In 2007, the Ministries of Health of Central America and the Dominican Republic implemented the PAHO-CDC Generic Protocol for Influenza Surveillance to identify influenza among severe acute respiratory infection (SARI) cases (defined as sudden onset fever >38 °C, cough or sore-throat, and shortness of breath or difficulty breathing requiring hospitalization) [23]. Cases were tested for influenza through immunofluorescence and real-time reverse transcription-polymerase chain reaction. Starting April 2009, countries also documented laboratory-confirmed influenza A(H1N1)pdm09 SARI cases that died in hospital within two weeks from illness onset. We used hospital-based surveillance to identify laboratory-confirmed influenza deaths in Costa Rica, El Salvador, Guatemala, Honduras, Nicaragua, Panama and Dominican Republic. SARI decedents were identified through event-based surveillance and outbreak response activities. Upon identifying potential cases, health authorities reviewed clinical records and autopsy reports to determine if decedents had indeed met the SARI case-definition.

We defined women of reproductive age as those aged between 15–49 years. We categorized pregnant women who developed respiratory illness before delivery as “pregnant” and those who developed respiratory illness within 42 days of delivery as “postpartum” cases. We categorized a case as obese if such a diagnosis was recorded in their medical record or if height and weight information were available and the body mass index exceeded 30 kg/m^2^.

### Data collection

We collected data using standardized questionnaires, from May 2009 to June 2010. Health authorities systematically obtained demographic (e.g. age and sex) and clinical information (e.g. date of illness onset, health seeking behavior, treatment with oseltamivir, history of pre-existing medical conditions, symptoms, signs, laboratory, radiology, and pathology findings) from SARI decedents associated with influenza A(H1N1)pdm09 from clinical records (by reviewing the medical records of hospitals, outpatient clinic records and referral sheets which were available) and obtaining information from relatives, and surveillance data.

### Ethics

The review of the clinical records was performed in the context of the pandemic outbreak for the characterization of the first 100 cases, following recommendations of WHO. For the emergency, at that moment, was no required approval for IRB. Approvals by Ministries of Health consisted of administrative permission to access medical records. The review of medical records was performed and with the accompaniment of officials of the Ministries of Health. No patient identifiers were received or used for analysis.

### Analyses

We summarized demographic and clinical characteristics of decedents associated with influenza A(H1N1)pdm09 using proportions to compare both, as the participation of each country and as the age groups, with variables of interest (ie. Percentage of pregnant and postpartum women, underlying medical conditions and treatment). We stratified the analyses into three age groups (i.e. 0–18, 19–64 and ≥65 years). We conducted Chi-square, t-tests and analysis of variance tests for comparisons when appropriate. We eliminated missing data from the calculations, buy declared in the results section.

We verify the check list for observational studies from the STROBE Statement.

## Results

### Epidemiologic and demographic case characteristics

**Fig. 1:**
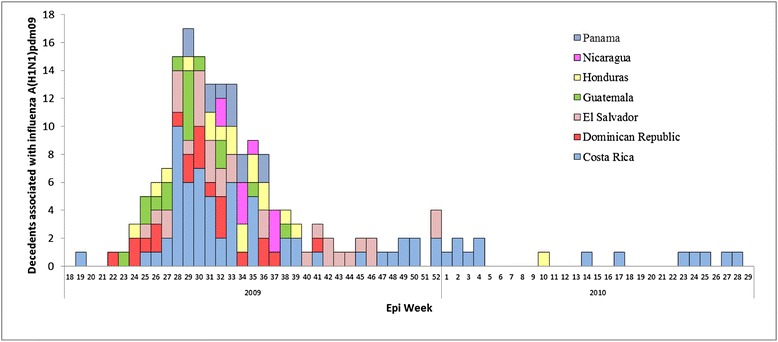
Number decedents associated with influenza A(H1N1)pdm09 by epidemiological Week, in Central America, 2009-2010

During epidemiological week (EW) 19 in 2009 through EW 25 in 2010, we identified 183 SARI decedents who tested positive for influenza A(H1N1)pdm09 in seven countries (Belize did not identify any influenza A(H1N1)pdm09 deaths). The first case was identified in Costa Rica during EW 19. Influenza A(H1N1)pdm09 deaths peaked during EW 30 (Fig. 1). Forty percent (74 cases) of the 183 decedents were identified in Costa Rica, 17 % (32 cases) in El Salvador, 11 % (21 cases) in Dominican Republic, 10 % (18 cases) in Honduras, 9 % (17 cases) in Guatemala, 7 % (12 cases) in Panama, and 5 % (9 cases) in Nicaragua (Table 1).

**Table 1:** Demographic characteristics of decedents associated with influenza A(H1N1)pnd09 in Central America, 2009-2010

Country	Decedents for influenza H1N1pnd09			General population (2009)	
	Number of cases	Median age in years (IQR)	Pregnant and postpartum women cases (%)	Percentage of pregnant woman in the general population by country^a^	Total country population (per million inhabitants)^b^
Costa Rica	74	41 (26–55)	4 (5 %)	1.6 %	4.6
Dominican Republic	21	25 (20–36)	5 (24 %)	2.2 %	9.8
El Salvador	32	15 (1–39)	6 (19 %)	2.1 %	6.1
Guatemala	17	34 (24–38)	5 (29 %)	3.3 %	14.0
Honduras	18	26 (3–42)	5 (28 %)	2.7 %	7.4
Nicaragua	9	30 (2–44)	1 (11 %)	2.4 %	5.7
Panama	12	23 (2–51)	1 (8 %)	2.1 %	3.5
All	183	32 (17–46)	27 (15 %)	2.3 %	51.1

The World Bank. Indicators. Birth rate, crude. Data section. Web page. Available in: http://data.worldbank.org/indicator/SP.DYN.CBRT.IN ^a^Calculated using the annual birth rate for 2009^a,b^

The median age among cases was 32 years (interquartile range: 18–46 years). Children aged <5 years represented 16 % (29 cases) and persons aged 15–44 years represented 48 % (88 cases) and persons aged >65 years represented 9 % (17 cases) of the 183 deaths. El Salvador had the lowest median age (15 years) and Costa Rica the highest (41 years) among decedents (Table 1). In El Salvador, cases aged <5 years were most prevalent (45 %) while in Guatemala cases aged 35–44 years (35 %).

Seventeen (9 %) of 183 cases had a history of smoking. Among 112 cases (61 %) with a pre-existing medical condition, 27 (15 %) were obese, 23 (13 %) had diabetes, 20 (11 %) had asthma, 15 (8 %) had other chronic metabolic diseases, 10 (5 %) had chronic obstructive pulmonary disease, 11 (6 %) had seizure disorder, and 7 (4 %) had cerebral palsy. Underlying medical conditions were identified in 38 % of cases <5 years old, in 61 % of cases aged 5–59 years old, and in all cases with 60 or more years old (Table 2). Among cases aged <18 years, seizure disorders (reported among 7 of 46 cases [15 %]) and asthma (6 cases [13 %]) were the most common pre-existing medical conditions. Among cases aged 18–64 years, obesity (24 of 120 cases [20 %]) and diabetes mellitus (19 cases [16 %]) were the most common. Among cases aged ≥65 years, diabetes mellitus (4 cases of 17 [24 %]) was the most common pre-existing medical condition. Cases with underlying medical conditions were older (mean age 37 years) than those without underlying medical conditions (mean age 26 years) (*p* < 0.01).

**Table 2:** Decedents associated with influenza H1N1pnd09. Pre-existing medical conditions in age risk groups and pregnant and postpartum women. Central America and Dominican Republic 2009–10

Underlying medical condition	All		<5 years		5 - 59 years		60+ years		Pregnant or postpartum	
	*N* = 183		*n* = 29		*n* = 134		*n* = 20		*n* = 27	
	Cases	%	Cases	%	Cases	%	Cases	%	Cases	%
Obesity	27	15 %	0	0 %	23	17 %	4	21 %	2	7 %
Diabetes Mellitus	23	13 %	0	0 %	16	12 %	7	37 %	2	7 %
Asthma	20	11 %	2	7 %	15	11 %	3	16 %	5	18 %
Other chronic metabolic disease	15	8 %	1	3 %	9	7 %	5	26 %	0	0 %
Immunosuppression	15	8 %	2	7 %	10	7 %	3	16 %	1	4 %
Chronic lung disease	10	5 %	0	0 %	8	6 %	2	11 %	2	7 %
Chronic seizures	11	6 %	3	10 %	8	6 %	0	0 %	0	0 %
Chronic cardiac disease	7	4 %	1	3 %	5	4 %	1	5 %	3	11 %
Cerebral palsy	7	4 %	1	3 %	6	4 %	0	0 %	0	0 %
At least one chronic condition	112	61 %	11	38 %	82	61 %	19	100 %	14	50 %

Percentages from the total of each category

One hundred and one (55 %) of 183 the decedents were female. Of the 101 female decedents, 61 (60 %) were women of reproductive age. Thirty-three percent (20 cases) of women of reproductive age who died were pregnant, and 7 (12 %) were in their puerperium. El Salvador reported that all women of reproductive age who died were maternal deaths, Guatemala reported 5 (71 %) of 7, and Honduras reported 5 (63 %) of 8 were maternal deaths.

Nearly half (48 %) of the pregnant women were in their third trimester, 24 % in the second trimester, and 24 % did not have a gestational age recorded. Fourteen (52 %) of 27 maternal deaths had other underlying medical condition (i.e. 5 (18 %) had asthma, 3 (11 %) had cardiac disease, 2 [7 %] had obesity, 2 [7 %] had chronic lung disease, 2 [7 %] had diabetes and 1 [4 %] AIDS) (Table 2). The proportion of pre-existing medical conditions between maternal deaths and cases without pregnancy were similar, except for the proportion with chronic cardiac disease which was higher among maternal deaths (11 % [3/27] cases) when compared to non-pregnant women (3 % [4/156], *p* = 0.03). The average age of maternal deaths was 29 years.

### Clinical, laboratory, radiology, and pathology characteristics

One hundred and fifty-one (82 %) of 183 decedents had a history of fever before their first contact with a clinic, but only 85 (46 %) had documented fever during admission. Dyspnea was reported in only 124 (68 %) of cases during admission.

Seventy five (41 %) of 183 cases had a chest x-rays (CXR) during their illness, of which 71 % had consolidation 53 % had interstitial findings. Five (28 %) of the 18 cases with interstitial infiltrates subsequently developed consolidation during hospitalization. Sixteen (9 %) of the 183 cases had autopsy reports, of which 11 (69 %) had cardiomegaly, 5 (31 %) intra-alveolar hemorrhage, 4 (25 %) neutrophilic bronchopneumonia, and 4 (25 %) cerebral edema.

### Timing of health seeking and oseltamivir use

Decedents sought care a median of 4 days (IQR 1–5 days) after symptom onset. The median duration between health seeking and death was 14 days (IQR 6–17 days). There was no statistically significant difference in the average amount of time elapsed between symptom onset and health seeking by age groups (*p* = 0.4), or country (*p* = 0.2). Overall, one hundred nineteen (65 %) out of the 183 cases received treatment with oseltamivir, (Table 3) but there was variability in the proportion treated depending on the country where they lived (range 25–89 %). Of the 119 that received oseltamivir, only 10 (5 %) received it within the first 48 h of onset of symptoms, and 21 (11 %) within the first 72 h (Table 3). Twenty (74 %) of 27 pregnant / postpartum women received oseltamivir (4 cases did not receive oseltamivir, and 4 had no treatment data). Oseltamivir use among pregnant /postpartum women was significantly higher compared to women who were not pregnant (OR = 10 [95 % CI 3–32]) but there was no difference between days from hospitalization and oseltamivir treatment (*p* = 0.9).

**Table 3:** Treatment of influenza A (H1N1)pnd09 in severe acute respiratory case-patients prior to death in Central America 2009-2010

Treatment	All *N* = 183		Age groups						Pregnancy or postpartum *n* = 27	
			<5 years *n* = 29		5–59 years *n* = 133		+60 years *n* = 21			
	Cases	%	Cases	%	Cases	%	Cases	%	Cases	%
Acute respiratory distress syndrome^a^	134	73	20	69	97	73	17	81	17	63
Intensive care admission^a^	113	62	18	62	84	63	11	52	15	56
Mechanical ventilation^a^	134	73	21	72	96	72	17	81	15	56
Oseltamivir	119	65	13	45	93	70	13	62	20	74
In first 48 h of symptoms onset^b^	10	5	1	8	8	6	1	8	0	0
In first 72 h of symptoms onset^b^	21	11	3	23	17	13	1	8	2	7

^a^Percentage of total category. ^b^Percentage who received oseltamivir

Fifty five percent (100/183 cases) were also tested through immunofluorescence for other respiratory virus. Two cases also were positive for respiratory syncytial virus, 1 for influenza B, 1 for adenovirus, 1 for parainfluenza virus. Twenty-three percent (43 cases) were tested for bacteremia through blood cultures; 1 was positive for *Streptococcus pneumoniae*.

## Discussion

Decedents infected with influenza A(H1N1)pdm09 were frequently young, pregnant, or had other pre-existing medical conditions. Unlike seasonal influenza deaths, which predominately occur among older adults [24, 25], the median age of those who died with influenza A(H1N1)pdm09 infections was 32 years. More than half had a pre-existing medical condition. In addition, 15 % of all decedents with influenza A(H1N1)pdm09 in Central America were reported as maternal deaths, more than twice the 2009 prevalence of pregnancy among study countries (6.5 %) [26]. The proportion of influenza A(H1N1)pdm09 maternal deaths was more than twice the global estimates (5–7 %) [27–30]. It could be effect of relevance of maternal mortality for these countries.

Such findings suggest that the influenza pandemic disproportionately affected pregnant women and mothers during their puerperium and consider targeting this group during future epidemic/pandemic periods with risk communications, early antiviral treatment, and vaccination [31].

Costa Rica had the higher proportion of deaths (40 %); this could be explained by diagnosis skills and access to health services in this country.

Although most influenza A(H1N1)pdm09 case-patients were treated with oseltamivir (65 %), less than 6 % received oseltamivir within 48 h of symptom onset when the antiviral are thought to be most effective [32–34]. We found no evidence that non-pregnant persons at high risk for complications [5–7, 9, 14, 19, 35] were more likely to receive timely oseltamivir than other cases. Indeed, there was a wide variability among the proportion of case-patients who were treated with oseltamivir depending on their country of residence. Such findings raise questions about the accessibility to timely oseltamivir stockpiles, physicians experience using oseltamivir during routine clinical care, and direct medical costs associated with oseltamivir in the American tropics. Our findings underscore importance of exploring the timely use of oseltamivir in middle and low income countries.

This study has important limitations. We assumed that all SARI decedents identified by health authorities comprised the majority of laboratory-confirmed influenza A(H1N1)pdm09 deaths. It is likely that a proportion of persons with severe influenza A(H1N1)pdm09 illness may have remained unidentified or untested and that these persons could have had different demographic and clinical characteristics than those identified through surveillance. For example, it is possible that cases with pre-existing medical conditions may have been preferentially tested for influenza A(H1N1)pdm09 thus increasing the proportion of high risk laboratory-confirmed case-patients identified in our case-series. Also we have no access to socioeconomic status of cases.

## Conclusions

Our study demonstrates the utility of influenza surveillance which provided valuable data to the epidemiology of influenza A(H1N1)pdm09 during 2009–2010. Our findings suggest that decedents associated with influenza A(H1N1)pdm09 were frequently young persons with prevalent pre-existing medical conditions. Our data suggest that few cases received oseltamivir within 48 h of treatment, highlighting challenges with the use of antivirals during periods of epidemic activity in the region. Based on these data, current national treatment protocols urge clinicians prioritize individuals with preexisting medical conditions such as pregnancy, asthma, and diabetes for timely oseltamivir treatment. Future studies should explore the availability and accessibility of oseltamivir as well as the potential value for presumptive treatment of high-risk individuals during periods of epidemic influenza activity in tropical middle and low income countries.

## Additional file

Additional file 1:
**STROBE Statement—Checklist of items that should be included in reports of** ***cross-sectional studies*** **.** (DOC 87 kb)

## Abbreviations

AIDS: Acquired immune deficiency syndrome
CDC: Centers for Disease Control and Prevention
CXR: Chest X Rays
EW: Epidemiological week
H1N1pdm09: Corresponding to pandemic strain of influenza for 2009
IQR: Interquartile range
OR: Odds ratio
PAHO: Pan American Health Organization
SARI: Severe acute respiratory infection
WHO: World Health Organization
