# Supplementary material for: Demographic and clinical characteristics of deaths associated with influenza A(H1N1) pdm09 in Central America and Dominican Republic 2009–2010
Source: BMC Public Health. 2015 Jul 31;15:734. doi: 10.1186/s12889-015-2064-z (PMC4521479; doi:10.1186/s12889-015-2064-z)
Supplement: Additional file 1: — STROBE Statement—Checklist of items that should be included in reports of cross-sectional studies . (DOC 87 kb) [file 12889_2015_2064_MOESM1_ESM.doc]

STROBE Statement—Checklist of items that should be included in reports of ***cross-sectional studies***

|  | Item No | Recommendation |
| --- | --- | --- |
| **Title and abstract** | 1 | (*a*) Indicate the study’s design with a commonly used term in the title or the abstract  Title mention is about the demographic and clinical characteristics |
|  |  | (*b*) Provide in the abstract an informative and balanced summary of what was done and what was found  We abstracted our methods (description of demographics and clinical characteristics), and our main findings regarding to proportion of deaths for each country, for sex, age groups, pre-existing medical conditions and use of antivirals. |
| Introduction | | |
| Background/rationale | 2 | Explain the scientific background and rationale for the investigation being reported  We explain the risk factors for complications related to influenza described in the literature. In addition, we explain the recommendations from international agencies to fight against the influenza, and the strategies implemented by the countries to minimize the complications and deaths. |
| Objectives | 3 | State specific objectives, including any prespecified hypotheses  We did not had a hypothesis.  Our objective is description of the demographics, clinical characteristics, and oseltamivir treatment of persons who died with laboratory-confirmed influenza A(H1N1)pdm09 |
| Methods | | |
| Study design | 4 | Present key elements of study design early in the paper  We describe the case definitions and methods for the identifications of the cases. |
| Setting | 5 | Describe the setting, locations, and relevant dates, including periods of recruitment, exposure, follow-up, and data collection  We used hospital-based surveillance to identify laboratory-confirmed influenza deaths in Costa Rica, El Salvador, Guatemala, Honduras, Nicaragua, Panama and Dominican Republic. |
| Participants | 6 | (*a*) Give the eligibility criteria, and the sources and methods of selection of participants  We identified SARI decedents through event-based surveillance and outbreak response activities using the current case definitions from PAHO. Upon identifying potential cases, health authorities reviewed clinical records and autopsy reports to determine if decedents met the SARI case-definition. |
| Variables | 7 | Clearly define all outcomes, exposures, predictors, potential confounders, and effect modifiers. Give diagnostic criteria, if applicable  We used a standardized questionnaire to get the following variables: days of illness onset, health-seeking behaviour, treatment with oseltamivir, history of pre-existing medical conditions, symptoms, signs, laboratory, radiology, and pathology findings. |
| Data sources/ measurement | 8* | For each variable of interest, give sources of data and details of methods of assessment (measurement). Describe comparability of assessment methods if there is more than one group  We used the data as data source the medical records. We defined death related to influenza, age groups, and women of reproductive age, postpartum cases and obesity. |
| Bias | 9 | Describe any efforts to address potential sources of bias  We tried to identify all possible cases. |
| Study size | 10 | Explain how the study size was arrived at  We included all identified cases |
| Quantitative variables | 11 | Explain how quantitative variables were handled in the analyses. If applicable, describe which groupings were chosen and why  We used a table, in which every row had a case.  We stratified the analyses into three age groups using the recommendations from Advisory Committee on Immunization Practices for complications related to influenza. |
| Statistical methods | 12 | (*a*) Describe all statistical methods, including those used to control for confounding  We summarized proportions to compare the participation of each country and the age groups, with percentage of pregnant and postpartum women, pre-existing medical conditions, and treatment. |
|  |  | (*b*) Describe any methods used to examine subgroups and interactions  We stratified the analyses into three age groups. We conducted Chi-square, t-tests and analysis of variance tests for comparisons when appropriate. |
|  |  | (*c*) Explain how missing data were addressed  We eliminated missing data from the calculations, buy declared in the results section. |
|  |  | (*d*) If applicable, describe analytical methods taking account of sampling strategy  Not applicable |
|  |  | (*e*) Describe any sensitivity analyses  We did not perform sensitivity analysis |
| Results | | |
| Participants | 13* | (a) Report numbers of individuals at each stage of study—eg numbers potentially eligible, examined for eligibility, confirmed eligible, included in the study, completing follow-up, and analysed  We described that, during epidemiological week (EW) 19 in 2009 through EW 25 in 2010, we identified 183 SARI decedents who tested positive for influenza A(H1N1)pdm09 in seven countries. We also described the number of pregnant women. |
|  |  | (b) Give reasons for non-participation at each stage  Not applicable |
|  |  | (c) Consider use of a flow diagram |
| Descriptive data | 14* | (a) Give characteristics of study participants (eg demographic, clinical, social) and information on exposures and potential confounders  We described proportions of cases by country, age groups, pregnancy, pre-existing medical conditions |
|  |  | (b) Indicate number of participants with missing data for each variable of interest  We indicate missing data for gestational age for pregnant women, and cases without chest x rays information. |
| Outcome data | 15* | Report numbers of outcome events or summary measures  Not applicable |
| Main results | 16 | (*a*) Give unadjusted estimates and, if applicable, confounder-adjusted estimates and their precision (eg, 95% confidence interval). Make clear which confounders were adjusted for and why they were included  Not applicable. We only describes proportions. |
|  |  | (*b*) Report category boundaries when continuous variables were categorized  We used age groups as follows: 0–18, 19–64 and ≥65 years. We consider obese if body mass index exceeded 30kg/m2. |
|  |  | (*c*) If relevant, consider translating estimates of relative risk into absolute risk for a meaningful time period  No applicate. |
| Other analyses | 17 | Report other analyses done—eg analyses of subgroups and interactions, and sensitivity analyses  We performed analysis for age groups and pre-existing medical conditions. |
| Discussion | | |
| Key results | 18 | Summarise key results with reference to study objectives  In the first paragraph of Discussion section, we describe the main prevalent groups and the proportion of pregnant women related to influenza H1N1pmd09 |
| Limitations | 19 | Discuss limitations of the study, taking into account sources of potential bias or imprecision. Discuss both direction and magnitude of any potential bias  We explained the limitations, ie. we assumed that all SARI decedents identified by health authorities comprised the majority of laboratory-confirmed influenza A(H1N1)pdm09 deaths. |
| Interpretation | 20 | Give a cautious overall interpretation of results considering objectives, limitations, multiplicity of analyses, results from similar studies, and other relevant evidence  We interpret our results and recommendations in conclusions section. |
| Generalisability | 21 | Discuss the generalisability (external validity) of the study results  We discussed the use of oseltamivir in priority risk groups. |
| Other information | | |
| Funding | 22 | Give the source of funding and the role of the funders for the present study and, if applicable, for the original study on which the present article is based  At the end of the manuscript, we included the financial disclosure. |

*Give information separately for exposed and unexposed groups.

**Note:** An Explanation and Elaboration article discusses each checklist item and gives methodological background and published examples of transparent reporting. The STROBE checklist is best used in conjunction with this article (freely available on the Web sites of PLoS Medicine at http://www.plosmedicine.org/, Annals of Internal Medicine at http://www.annals.org/, and Epidemiology at http://www.epidem.com/). Information on the STROBE Initiative is available at www.strobe-statement.org.
